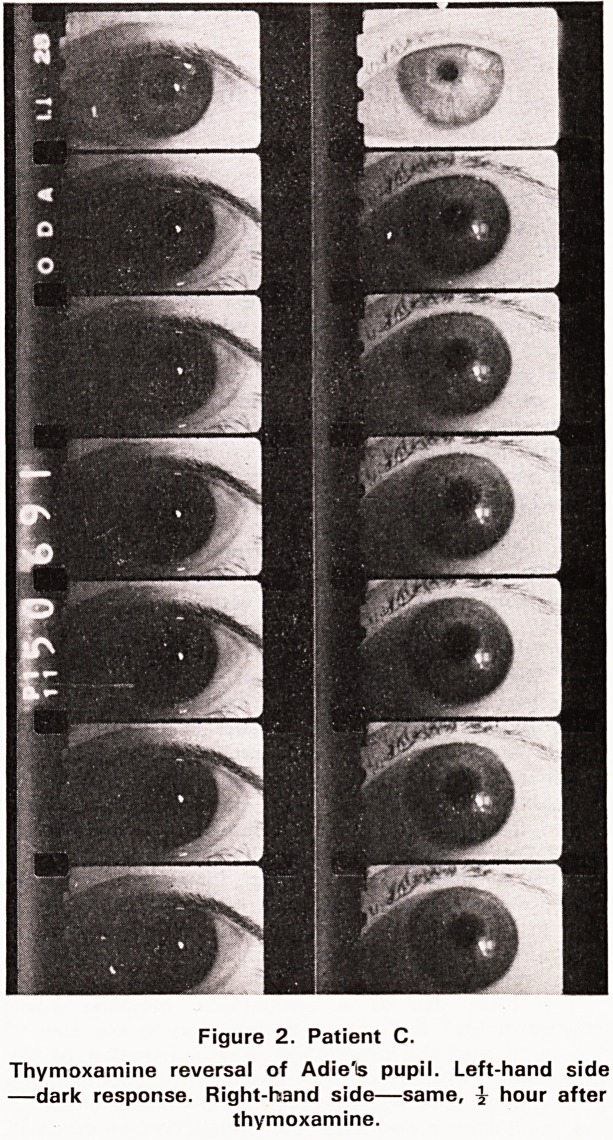# Adie's Syndrome

**Published:** 1975-04

**Authors:** Vincent Marmion

**Affiliations:** Bristol


### Adie's Syndrome

**Vincent Marmion, D.O.M.S., M.R.C.P.E., F.R.C.S.Ed.**
Bristol

On the whole, the fascinating pupillary abnormality which comes under the title of Adie's Syndrome is not of significant embarrassment to the patient. Apart from the cosmetic appearance, the principal complaint is that of photophobia. Associated symptoms, particularly those which would come under the category of gastric "dumping", were probably originally thought to be unrelated to the Adie's Syndrome. With the advent of evidence on the pathological basis of Adie's Syndrome by Harriman and Garland (1969), a more rational basis for the association of the two conditions became apparent.

In 1969, therefore, the late Dr. Campbell and myself undertook a review of some twelve patients who were known to be suffering from Adie's Syndrome (1932). Of these, nine out of the twelve were left sided, concurring with the observation of Foster Moore (1924). The earliest stage of onset, as far as we could establish was six years of age, and the eldest forty two. There was one male patient and, in one patient, there was a suggestion of old chorio-retinitis. In one other patient there was a suggestion of some acute febrile illness prior to the onset of the disease. All patients had shown a response to a weak solution of topical mecholyl 0.5 per cent, and all patients were tested to see if there was a response to a weak alpha sympathetic blocking agent thymoxamine (Opilon). The use of stellate ganglion block for Adie's Syndrome has previously been described (Russell 1956). It is also of interest to note that the advent of Adie's Syndrome in one patient followed the taking of Dexadrine for slimming.

The local use of a sympathetic blocking agent (thymoxamine) has shown a reduction in the size of the pupil and the production of a restoration of the direct and indirect light responses. The accompanying photographs (see figures 1 and 2) show the effect on the pupil before and after the installation of drops in four patients. These photographs were taken with an infra-red camera in absolute darkness. It can be seen that the pupil in several of the cases, dilates very slightly during the brief period of observation.

Sympathetic blocking agents have been a great help to some patients in producing a tolerable myosis without any accommodative change. They have been unaffected by light and the control of the pupil has, on many occasions, lasted for quite some period of time. It is perhaps significant that the syndrome with its basis in a degeneration of the parasympathetic autonomic fibres in the ciliary ganglion is so much more frequent in female than male subjects.

Four out of the twelve patients suffered from indigestion and in three of these the rate of gastric emptying after a barium meal was 5½, 6 and 8 hours, respectively. A fifth patient had considerable problems with micturition and the use of Opilon produced, at least for a while, a very considerable improvement in the frequency of micturition and the control of bladder function. The major problem in this patient was assessing the true value of this as there was a significant psychological overlay.

The conclusions from these observations suggest that the autonomic parasympathetic degeneration which is present in Adie's Syndrome may not be solely a localised condition. The basic pathology may, on occasions, have arisen from an acute febrile illness which has affected specifically this branch of the nervous system. Cases have been recorded of Adie's Syndrome occurring after acute febrile illnesses and also in association with chronic progressive polyneuropathy (Inokuchi et al 1972).

The use of sympathetic blocking agents is a relatively simple method of controlling the pupillary reaction and by local topical drops. These can be used in a concentration of either, 0.1 or 0.5 per cent. Systemic Opilon has not been tried for its effect on the pupil except in the one case of the woman who had problems with micturition. In this instance, the pupil did reduce in size and light reactions were restored. The use of Opilon systemically is not associated with any significant fall in blood pressure in those cases in which it was tried.

It would be of interest to follow those cases up further in which there is evidence of delayed gastric emptying to see if this can be quantified in its response to the administration of Opilon.

1. ADIE, W. J. 1932. Tonic pupils and absent tendon reflexes. Brain. *55.* 98.
2. FOSTER MOORE, R. 1924. The Physiology & Pathology of the Pupillary Responses. Trans. Oph. Soc. U.K. *44.* 38.
3. HARRIMAN, W. G. September 1968. The pathology of Adies Syndrome. Brain *91.* 401-18.
4. INOKUCHI, T. et al 1972. Case of chronic progressive polyneuropathy associated with Adies Syndrome. Clinical neurology (Tokyo) *12.* 355. 62.
5. RUSSEL, G. F. M. 1956. Pupillary changes in the Holmes Adie Syndrome. J. Neurol Neurosurg and Phychiat. *19.* 289.

Figure 1. Patient P.

Thymoxamine reversal of Adie's pupil. Left-hand side —dark response. Right-hand side—same, ½ hour after thymoxamine. Ptosis present after treatment.

Figure 2. Patient C.

Thymoxamine reversal of Adie's pupil. Left-hand side —dark response. Right-hand side—same, ½ hour after thymoxamine.

## Figures and Tables

**Figure 1. f1:**
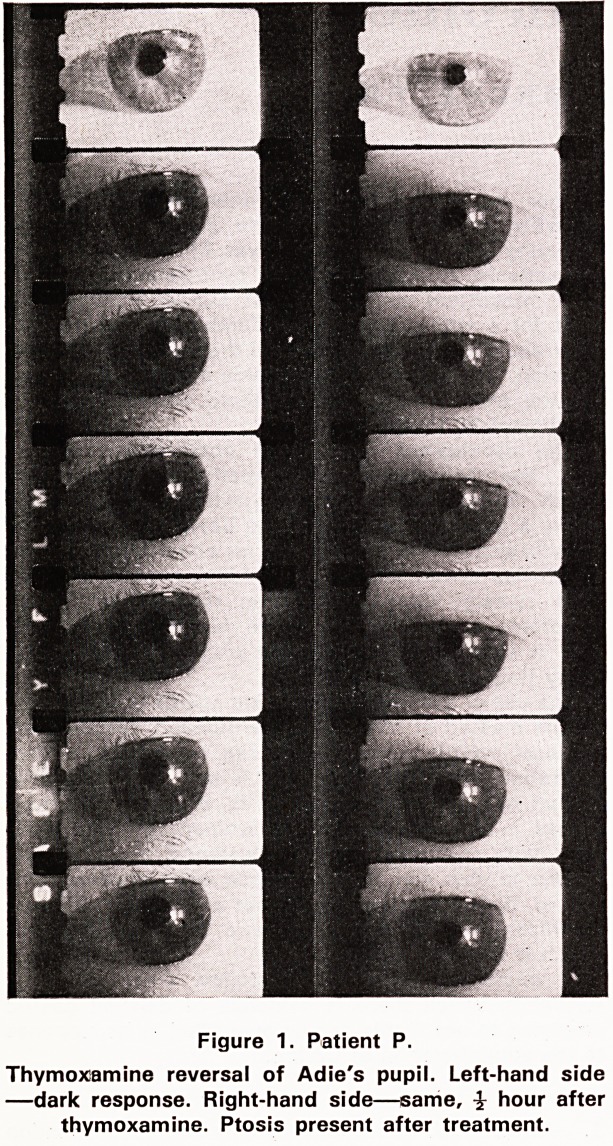


**Figure 2. f2:**